# Phytochemicals and bioactive constituents in food packaging - A systematic review

**DOI:** 10.1016/j.heliyon.2023.e21196

**Published:** 2023-10-24

**Authors:** Shahida Anusha Siddiqui, Sipper Khan, Mohammad Mehdizadeh, Nur Alim Bahmid, Danung Nur Adli, Tony R. Walker, Rosa Perestrelo, José S. Câmara

**Affiliations:** a Technical University of Munich Campus Straubing for Biotechnology and Sustainability, Essigberg 3, 94315, Straubing, Germany; b German Institute of Food Technologies (DIL e.V.), Prof.-von-Klitzing Str. 7, 49610, D-Quakenbrück, Germany; c Tropics and Subtropics Group, Institute of Agricultural Engineering, University of Hohenheim, 70593, Stuttgart, Germany; d Department of Agronomy and Plant Breeding, Faculty of Agriculture and Natural Resources, University of Mohaghegh Ardabili, Ardabil, Iran; e Ilam Science and Technology Park, Iran; f Research Center for Food Technology and Processing, National Research and Innovation Agency (BRIN), Gading, Playen, Gunungkidul, 55861, Yogyakarta, Indonesia; g Agricultural Product Technology Department, Universitas Sulawesi Barat, Majene, 90311, Indonesia; h Faculty of Animal Science, University of Brawijaya, Malang, East Java, 65145, Indonesia; i School for Resource and Environmental Studies, Dalhousie University, Halifax, Nova Scotia, B3H, 4R2, Canada; j CQM – Centro de Química da Madeira, Universidade da Madeira, Campus da Penteada, 9020-105, Funchal, Portugal; k Departamento de Química, Faculdade de Ciências Exatas e da Engenharia, Universidade da Madeira, Campus da Penteada, 9020-105, Funchal, Portugal

**Keywords:** Consumer demand, Food packaging, Antimicrobial packaging, Consumer behaviour, Environmental concerns, Antioxidant compounds, Sensory

## Abstract

Designing and manufacturing functional bioactive ingredients and pharmaceuticals have grown worldwide. Consumers demand for safe ingredients and concerns over harmful synthetic additives have prompted food manufacturers to seek safer and sustainable alternative solutions. In recent years the preference by consumers to natural bioactive agents over synthetic compounds increased exponentially, and consequently, naturally derived phytochemicals and bioactive compounds, with antimicrobial and antioxidant properties, becoming essential in food packaging field. In response to societal needs, packaging needs to be developed based on sustainable manufacturing practices, marketing strategies, consumer behaviour, environmental concerns, and the emergence of new technologies, particularly bio- and nanotechnology. This critical systematic review assessed the role of antioxidant and antimicrobial compounds from natural resources in food packaging and consumer behaviour patterns in relation to phytochemical and biologically active substances used in the development of food packaging. The use of phytochemicals and bioactive compounds inside packaging materials used in food industry could generate unpleasant odours derived from the diffusion of the most volatile compounds from the packaging material to the food and food environment. These consumer concerns must be addressed to understand minimum concentrations that will not affect consumer sensory and aroma negative perceptions. The research articles were carefully chosen and selected by following the Preferred Reporting Items for Systematic Reviews (PRISMA) guidelines.

## 1 Introduction

The term phytochemical refers to plant chemicals with different compositions and properties. In plants, these substances perform various functions for defence and growth, such as attracting insects for pollination, protecting plants from pathogens, regulating growth and reproduction, controlling insects, and protecting plants against herbivores [1]. Health benefits and disease prevention can be achieved from dietary consumption of phytochemicals [2]. Plant foods, including vegetables, fruits, seeds, whole grains, and nuts, are the primary providers of phytochemicals [3]. Phytochemicals in plant foods minimise the occurrence of chronic disorders like heart attacks [4]. Over the past two decades, bioactive phytochemicals have been systematically investigated both *in vitro* and *in vivo*, providing a valuable understanding of mechanisms presumably contributing to disease prevention. There has been increasing interest in the impacts of food processing on the chemical composition of food in recent studies [[5], [6], [7]]. These compounds include phenolic compounds, carotenoids, phytosterols, glucosinolates, minerals, vitamins, enzymes, bacteriocins, and unsaturated fatty acids [8]. Over 5000 phytochemicals have been discovered in plant-based foods, differing in structure and composition [9]. Nevertheless, considerable research is being conducted in various world areas regarding phytochemicals' chemical structure and biological function. Plants are protected by phenolic compounds in fruits and vegetables from environmental stressors like pollutants, parasites, and other abiotic stresses [10].

Interest in studying, designing, and manufacturing functional bioactive ingredients and pharmaceuticals has grown worldwide. As customers become more knowledgeable about the connection between food, health, and disorders, the use of plant-based bioactive ingredients has risen. Traditionally and contemporary medicines can all be created from medicinal plants [11]. They are also the primary bioresource for pharmaceuticals, dietary additives, and chemical components for manufactured medicines. Several clinical and animal tests indicate that the consistent intake of whole grains, fruits, and vegetables helps prevent oxidative damage-related diseases [[12], [13], [14], [15]].

Using natural plant compounds in packaging materials to enhance their functional qualities is referred to as the link between phytochemicals and the food industry. Plants contain bioactive substances that have antioxidant, and antimicrobial properties, with impact on human health. Manufacturers can design packaging materials that can aid in maintaining the quality and safety of food for extended periods by adding these chemicals into food packaging. Due to the growing desire for sustainable and environmentally friendly packaging options that can provide extra health benefits; thus, this strategy has attracted more and more interest in recent years.

Plants produce phytochemicals by metabolising sugars, acids, and polysaccharides [16]. They are also known for their antioxidant, antimicrobial, antiproliferative, antidiabetic, neuroprotective and antihypertensive properties, among others. Numerous studies have been published on plant antimicrobial-antiviral, antifungal, and antibacterial properties [[17], [18], [19], [20]]. From production to consumption, food packaging is an essential process in the modern food production system since it helps maintain food quality [21,22]. In response to societal needs, food packaging has become increasingly innovative. Packaging needs to be developed based on manufacturing practices, marketing strategies, consumer behaviour, environmental concerns, and the emergence of new technologies, particularly bio and nanotechnology.

Consequently, packaging should extend the shelf-life of food, ensure the safety and quality of the food product, enhance consumer health, and minimise environmental impact. As a result, the efficient marketing of active and intelligent packaging systems depends on potential customers' approval. Therefore, this critical systematic review aims to discuss phytochemicals and bioactive compounds in food packaging.

## 2 Methodology

### 2.1 Eligibility criteria, articles search strategy and dataset development

**Fig. 1:**
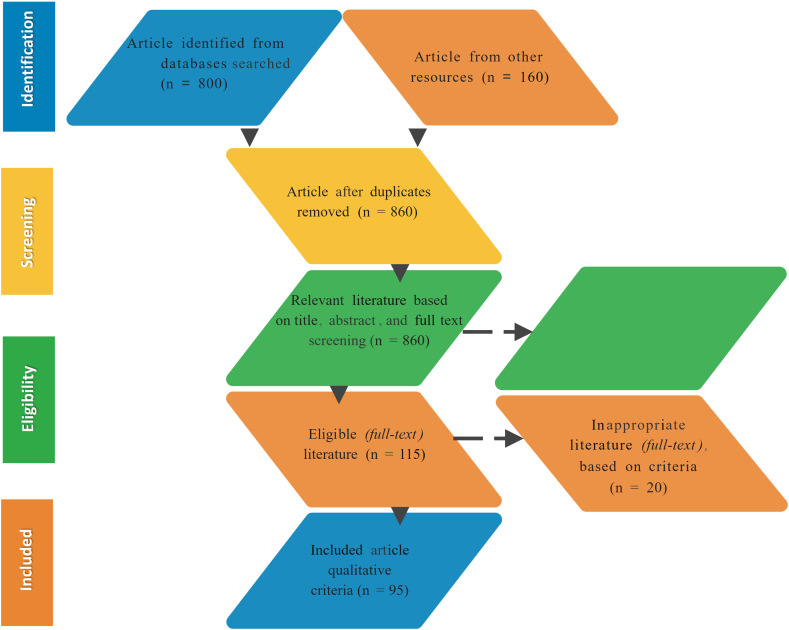
Diagram flow of article selection in consumer studies focused on phytochemicals and bioactive constituents in food packaging.

We applied the following inclusion in this review by following population, intervention, comparators, outcomes, and study design (PICOS) as follows: (1) Consumers; (2) phytochemicals; (3) consumer studies focused on phytochemicals and bioactive constituents in food packaging; (4) articles consistently written in English and published after being peer reviewed. After careful evaluation, a raw dataset that reported consumer studies focused on phytochemicals and bioactive constituents in food packaging was constructed and extracted. The articles were carefully chosen and selected following the Preferred Reporting Items for Systematic Reviews (PRISMA) guidelines [23]. Published articles were extracted into Mendeley references manager (https://www.mendeley.com/) with the following criteria: (1) name of the author; (2) publication year; (3) year of study; (4) type of alternative protein used; and (5) results. Initially, 860 results were achieved through the Science Direct database (https://www.sciencedirect.com/). From these, 425 articles were excluded due to not being related to the effects of phytochemicals and bioactive constituents of food packaging on consumers. One hundred and fifty-eight articles were excluded since they reported unrelated parameters. One hundred eighty-two articles were excluded because they did not note bioactive constituents or phytochemicals. Finally, 95 articles remained for systematic review for consumer studies focused on phytochemicals and bioactive constituents in food packaging (Fig. 1). The algorithm search key for the published article was set from 2007 to 2022, using the MESH terms (“behaviour”) AND (“consumers”) AND (“packaging” OR “foods” OR “bioactive” OR “phytochemicals”).

## 3 Phytochemicals and bioactive constituents in active and intelligent food packaging systems

**Fig. 2:**
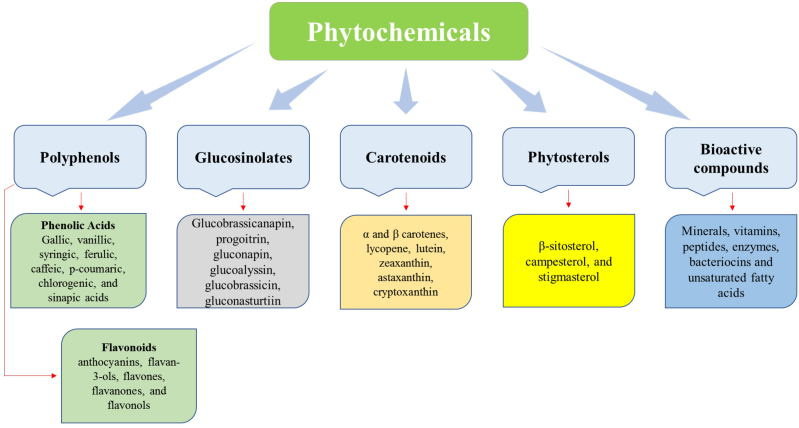
Phytochemical compounds involved in food packaging systems.

The need for clean-label foods is growing today. Therefore, consumers' demand for safe ingredients and concerns over the harmful nature of synthetic additives has prompted food manufacturers to seek safer natural alternatives. Food packaging plays an essential role in contemporary food production, protecting against physical, chemical, and biological hazards throughout the production process [24]. Packaging has a protecting role also during transport, storage, and handling until its use. Essentially, the packaging extends shelf life and maintains more stable food products' nutritional value and quality. Increasing living standards and concerns about the quality and safety of food have prompted people to seek biodegradable, recyclable, and safe packaging materials to prevent chemicals from entering their food supply [25]. Both production and consumption of packaging materials made of plastic have increased significantly following the application of plastics for packaging. It offers a variety of benefits, including simplicity, cost-effectiveness, lightness, durability, and flexibility, and is widely utilised for packaging foods [26]. Plastics have severe environmental consequences, despite improving the food shelf life since they are rarely recyclable and biodegradable, especially in single-use packaging. Researchers have attempted to integrate phytochemical extracts within natural biopolymers to eliminate chemical contaminants in food as a replacement for plastic packaging. Food packaging involves using phytochemicals found in many plants, vegetables, fruits, and herbs. Fig. 2 shows the most critical phytochemicals used in food packaging systems. The main phytochemical compounds involved in food packaging systems are discussed in the following sections.

### 3.1 Polyphenols

Phenolic compounds found in plant-based foods have received particular consideration. Phytochemically, polyphenols are structurally related to phenolic compounds and are among plants' most abundant natural substances. Based on their chemical structure, polyphenols can be grouped into two categories: flavonoids and non-flavonoids. The flavonoids constitute the most important group, presenting a common diphenylpropane structure of the C6–C3–C6 type, consisting of two aromatic rings typically linked by an oxygenated heterocycle of three carbon atoms. According to the variations of the heterocyclic ring, flavonoids can be subdivided into several subclasses, the most important being represented by flavonols, flavones, flavanones, isoflavones, flavonoids and anthocyanins [27]. The non-flavonoids included the phenolic acids, the stilbenes and the lignans as the main sub-classes. This wide variety of compounds differs significantly in their bioavailability, structure, and biochemical properties [28]. However, they have garnered considerable popularity due to their use in the food industry. In addition to their antioxidant properties, they may possess essential antibacterial effects, which are not yet fully understood [29]. Several mechanisms are involved in this process, including changes in cell membrane fluidity, changes in intracellular processes connected to phenolics binding to enzymes, and a decrease in cell wall stability due to membrane interactions [30]. Many plants contain polyphenols, but their potential as natural alternatives to conventional preservation in food is quite exciting. Polyphenols can inhibit bacterial growth and fungi, indicating their importance for food production. Understanding polyphenols' antimicrobial properties is vital because their efficacy is influenced by pathogen sensitivity and chemical composition [31].

Extracts from plants are often added to packaging products and incorporated into films. The phenolic compounds found in plants, especially polyphenols and flavonoids, work as antimicrobials and antioxidants. Active packaging containing phytochemicals prevents food contamination and decay without contacting foods directly and altering their nutritional value [32]. Extracts from plant sources containing polyphenol (flavonoids and non-flavonoids), and alkaloid compounds with antioxidant capacity can be used effectively as additives in food packaging [33]. One of the most widely used by-products in the food industry is bran, produced from grain processing. Bran is inexpensive, readily available, and has many beneficial properties (anti-inflammation and antioxidant) because it is rich in phenolic substances, minerals, and fibres [12]. Wang et al. [34] reported tea polyphenols as a promising approach for developing effective biodegradable active packaging films. As a result of their remarkable antioxidant and bactericidal properties, polyphenols have been used to enhance food packaging materials' structural and chemical properties. Researchers have recently investigated the possibility of grafting polyphenols with manufactured polymers to boost packaging performance, reduce chemical contaminants, and prevent losses of active substances [35].

### 3.2 Carotenoids

Carotenoids are pigments abundantly found in nature and made by different organisms, including bacteria, algae, and fungi [36]. Two types of carotenoids are found in nature: xanthophylls, a group of oxygenated compounds (lutein, cryptoxanthin, zeaxanthin, and fucoxanthin), and carotenes, pure hydrocarbon compounds (lycopene, α, and β-carotenes) [37]. Carotenoids' primary biological functions are to act as antioxidants and neutralise reactive oxygen species (ROS). They also have antimicrobial, antihyperglycemic, and anti-inflammatory properties, preventing heart and neurological diseases and enhancing immunity [38]. Due to their beneficial properties, carotenoids proved an appropriate substitute for synthetic ingredients, commonly associated with adverse effects [39]. These pigments are also helpful for colouring foods and enhancing their nutritional value [40]. Carotenoids are present in various vegetables and fruits, including tomatoes, carrots, watermelons, and some fish species, including salmon and crustaceans [41]. Food packaging biofilms also contain carotenoids such as α- and β-carotene and lycopene. β-Carotene can be incorporated into organic packaging films to provide an active component [42]. Stoll et al. [43] studied the effect of carotenoid extracts on polylactic acid films on sunflower oil preservation. Sunflower oil was protected from oxidation by β-carotene, lycopene, and bixin. Szabo et al. [44] demonstrated the effectiveness of carotenoids and phenolic compounds combined with tomato by-product extract in obtaining intelligent and active packaging using polyvinyl alcohol [45]. Lycopene belongs to the carotenoid group of phytochemicals applicable to active packaging [46].

### 3.3 Phytosterols

Plant sterols are substances with identical compositions and properties to cholesterol but from vegetable origin. Vegetables contain plant sterols, but human bodies do not produce them [47]. Phytosterols contribute to cell membranes' structural and functional health by maintaining their integrity and stability [48]. The interaction between phytosterols directly affects cell membranes and other essential functions. Generally, β-sitosterol, stigmasterol, and campesterol are the most common phytosterols [49]. The advantages of phytosterols led to an increased interest in using these compounds to develop foods containing phytosterols for the prevention and treatment of coronary disorders. Phytosterols are practical and safe options for preventing coronary vascular disease as functional food ingredients [48]. These ingredients can be found in many foods today. It is common for food manufacturers in developed countries to produce functional foods with elevated levels of sterols and stanols. Plant sterols and stanols can be incorporated into food forms without altering the taste or texture [50]. A wide variety of products currently include phytosterols, from bars, plant oils, juice, meat, soups, dairy and baked products [51,52].

Nevertheless, recent research has suggested that coatings made of phytosterols may be employed to improve the barrier qualities of food packaging materials. It was discovered that adding phytosterols to polyethylene films enhanced their capabilities as an oxygen barrier, potentially extending the shelf life of goods stored in these containers. More research is nonetheless required to ascertain the possible uses and restrictions of employing phytosterols as a food packaging material.

Fruits, legumes, grain crops, and seeds contain phytosterols and metabolites [53]. In terms of reducing blood cholesterol, phytosterols have been widely investigated. Plant stanol fatty acid esters were fortified into Benecol margarine in 1995, the first food enriched with phytosterols [47]. A wide variety of phytosterol-enriched functional foods have been produced and marketed worldwide over the years. Phytosterols' solubility and bioavailability might be influenced by other components involved in food preparation [54]. It is necessary to develop reliable and accurate techniques for extracting and determining phytosterols to assist the food industry and ensure the accuracy of nutritional labelling [47].

It should be noted that the type of packaging selected for preserving a phytosterol-enriched food may also influence its quality. Minimizing the exposure to light on the packaging of yoghurt beverages containing phytosterols could be feasible to maintain their nutritional properties [55]. Microencapsulation could be considered a mechanism that preserves the bioactive substances that contribute to the food composition required to ensure that phytosterols will not interfere with production or preservation [56].

Besides, phytosterols are primarily used as dietary supplements and food additives, with health benefit properties including its potential applications in managing cardiovascular health and cholesterol-lowering effects. It is well known the capacity of phytosterols as antioxidant and antimicrobial agents. However, to the best of our knowledge, there are no published studies that demonstrate the use of phytosterols in food packaging. As for other bioactive compounds, phytosterols can also be used in functional food packaging materials, namely as antioxidant, antimicrobial, and barrier agents. In this context its application in the development of biodegradable smart food packaging materials, for preserving food quality and safety, to make them more sustainable and environmentally friendly, would be an innovative approach with great industrial interest.

### 3.4 Glucosinolates

Secondary plant metabolites, glucosinolates, are derived primarily from the glucose and sulphur-containing compounds in dicotyledon plant species, with the most significant concentrations occurring in Brassicaceae [57]. These substances are readily accessible since these plants comprise many essential foods, such as fruits, herbs, vegetables, and oil crops [58]. Glucosinolate compounds have chemical stability and cannot induce biological function unless located inside the plant's subcellular structures. Glucosinolates are metabolised in vacuoles when plant cells are damaged by pathogens, harvest practices, food preparation, or consumption, in addition to thermal degradation [59]. A glucosinolate can be classified as aliphatic or aromatic based on its different side chains; currently, over 130 glucosinolates have been discovered [60]. Isothiocyanates are a class of bitter organic substances found in mustard oils; they are widely studied glucosinolate-derived compounds [61]. Glucosinolates and their metabolites have been proven to have health benefits, including reducing the risk of cancer, cardiovascular, inflammatory, and neurological disorders, controlling asthma and diabetes, and managing cholesterol levels [62,63]. Glucosinolates in fresh edible plant species have a bitter taste resulting from their enzymatic decomposition caused by myrosinase. This enzyme is found in the cytoplasm, whereas glucosinolates are reserved in the vacuole [64]. It is possible to have noticeable variations in the glucosinolate levels within the same species where cultivars can have significant quantity and quality variations. Glucosinolate levels differ across various parts of the same plant because they are tissue-specific [65].

Bahmid et al. [66], designed an active antimicrobial packaging based on the controlled release of allyl isothiocyanate (AITC) from mustard seed. They investigated the effect of fat content and particle size of ground mustard seeds on forming and releasing of AITC, and the underlying mechanisms were highlighted. In addition, Duda-Chodack et al. [67] reported on different groups of bioactive compounds that are used in smart and active packaging, namely as antimicrobial agents, including the AITC.

### 3.5 Other bioactive compounds

The use of other bioactive compounds (minerals, vitamins, peptides, enzymes, bacteriocins and unsaturated fatty acids) in packaging could be applied to various food systems and products, including grain-based foods, dairy foods, vegetables and fruits, meat-based foods, and seafood [68,69]. Vitamins, essential oils, minerals, lipids, and other bioactive compounds are crucial to human health. A vitamin-rich diet can benefit the immune system and antibody production and prevent eye, respiratory, and gastrointestinal infections. Similarly, inflammation-reducing properties are associated with vitamins and other bioactive compounds [70]. Vitamins, which provide an antioxidant function, may be included in the food production procedure since they defend themselves from oxidative damage [71]. The fundamental composition of proteins is composed of amino acid chains joined by peptide bonds.

Regarding food packaging, proteins are mainly used due to their outstanding physical and oxygen-blocking properties [72]. In addition, protein provides excellent bonding with polar compounds and prevents the escape of ingredients and bioactive compounds from the packaging system [73]. Accordingly, scientists have been developing bio-based packaging in the form of edible films and materials from bioactive compounds [74]. In food packaging systems, antimicrobial peptides can be considered adequate. They can provide significant inhibition of pathogen reproduction and infections with minimal quantities. Therefore, meat products containing these compounds exhibit decreased lipid oxidation [75].

Minerals, vitamins, peptides, enzymes, bacteriocins, and unsaturated fatty acids are examples of bioactive substances that can be used to create bio-based and edible packaging materials. Some of these substances, such as enzymes and bacteriocins, can be employed as functional or active components in packing materials. Antimicrobial peptides known as bacteriocins are produced by bacteria and can be utilised to prevent the growth of dangerous microorganisms in food packaging. On the other side, enzymes can be employed to improve the barrier characteristics or biodegradability of packing materials to improve their performance. For example, a biopolymer matrix made of proteins and peptides can be utilised to create edible packaging. Moreover, unsaturated fatty acids can enhance biopolymer matrices' flexibility and mechanical strength. The precise functions played by these bioactive substances in packaging materials depend on their chemistry, physics, and interactions with other elements of the packaging matrix.

Unsaturated fatty acids are typically employed as additives in packaging products since they effectively eliminate oxygen [76]. In today's world, antimicrobial packaging is crucial for food safety and quality, as it prolongs the storage life of food products and reduces the growth of bacteria on them. This practical packaging approach is now known as “active packaging”. Therefore, active peptide promotes food safety by improving the product's shelf life and enhancing the sensory characteristics of the food [77]. The popularity of this type of packaging is rising due to its capability to supply healthy, organic, and reliable foods to the market [52]. A systemic approach utilising intelligent and active packaging, such as enzyme-based packaging, can promote food safety and conservation while meeting changing cultural and economic requirements [78].

## 4 Consumer behaviour models for phytochemicals and bioactive agents used in food packaging

**Fig. 3:**
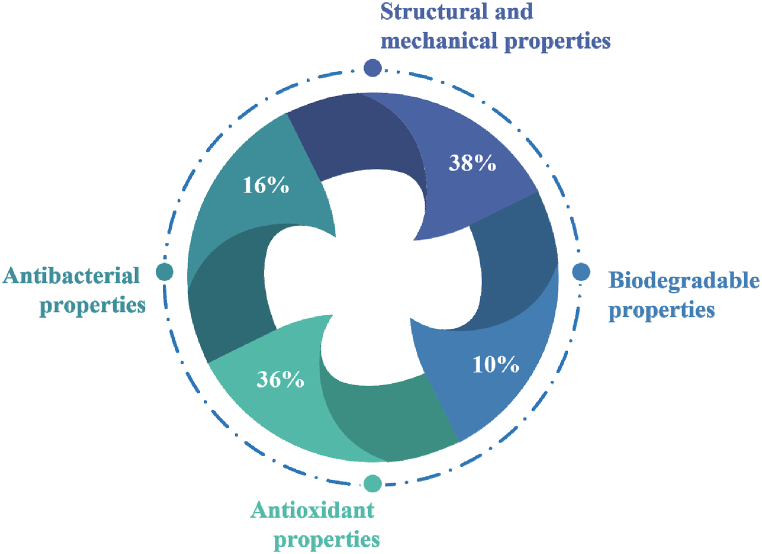
Consumer preference parameters in food packaging (Adapted from Geueke et al. [80]; Han et al. [81]; Trajkovska Petkoska et al. [82]).

One of the tasks of the food industry is to increase the shelf life of products, in particular, to create optimal packaging for food products, which should have antioxidant and antibacterial properties. For example, Narasagoudr et al. [79] describe the possibility of using bioactive chitosan and polyvinyl alcohol films induced by rutin to create food packaging. The study showed that adding rutin to the packaging improves the barrier to UV light, water vapour permeability (WVP), oxygen transmission rates (OTR), water resistance, water solubility and thermal properties. Adding rutin increases the density of the bioactive film mesh and enhances the antioxidant effect. Such bioactive films have shown intense antimicrobial activity against *Escherichia coli* and *Staphylococcus aureus* bacteria. After analysing the statistical data of the United States Food, and Food Drug Administration (US, FDA), it can be concluded that most food stakeholders use films with high antibacterial and antioxidant activity in their production (Fig. 3).

In some countries, adding polyvinyl alcohol (PVA) into food packaging films is allowed to increase the shelf life of food, which leads to options for optimising the composition and structure of the film. Thus, in the work of He et al. [83], a composite film of active PVA was developed, including pomegranate peel extract (PPE) and sodium dehydroacetate (SD). The study results showed that adding PPE to PVA film samples led to augmented antibacterial activity in relation to various bacterial cultures, which made it possible to include PPE and SD in PVA films for potential use as active packaging films or coatings.

According to the Global Food Safety Initiative, the main goal is to create an expanded food safety community to monitor food safety standards for businesses and provide access to safe food for people worldwide. Small enterprises also participate in the sustainable development of the Food Packaging industry [84]. A striking example is the work of Aziman et al. [85] where the authors considered ethanol extract and *Persicaria hydropiper* essential oil as biologically active additives and investigated their potential as antibacterial agents in the PVA film. According to the study, the integration of ethanol extract and essential oil in the PVA film also showed an antibacterial effect against *S. aureus*, which makes it possible to use this material in food packaging.

The use of nanotechnology in developing these products turned out to be another step-in advance in the food packaging industry. Based on the European Food Safety Authority data, including nanocellulose in the film increases the antimicrobial activity effect against various microbial cultures, such as Gram-positive *S*. *aureus* ATCC6538P and the Gram-negative *E. coli* ATCC8739. Ahankari et al. [86] studied nanocellulose (NC) and its multiple forms, including chemical and physical processing in biopolymers. They evaluated the effect on the characteristics of nanocomposites for use in food packaging. The review showed that the trends in *N*C-based materials research are promising for active packaging (AP) applications, including controlled release packaging (CRP) and adaptive packaging (AdP). As a result, the authors established assessments of the problems of eco-friendly packaging and grey areas that need improvement in the commercial use of described packaging material.

### 4.1 Food preservation

An essential component in the storage and transportation of food products is the preservation of the constancy of the composition of the food shell. Consumers from all over the world need to deal with the problem of external factors. The temperature and humidity of the environment greatly influence the quality of products, so the urgent task is to create food packages with maximum resistance to environmental conditions. One such solution was presented by Nešić et al. [87], who used polysaccharide-based materials. Using such materials is an environmentally safe technological solution that reduces dependence on fossil resources. Particular attention is paid to hemicelluloses, marine polysaccharides, bacterial exopolysaccharides, and their potential application in the latest trends in food packaging materials, including edible coatings, intelligent films, and thermally insulated aerogel packaging. In this context, Sharma et al. [88] studied various properties of AP-containing essential oils. Essential oil increases the UV barrier property and the surface hydrophobicity in AP while preserving foods by releasing antioxidant or antimicrobial agents. Since food packaging can be considered a passive barrier protecting products from various environmental factors, AP provides the possibility of interaction between the external environment and food products because the shelf life of food products is extended. Chemoactive packaging affects the chemical composition of the food product. Additionally, natural additives, such as essential oils, in active packaging can be used in films and coatings. It has been established that AP helps maintain temperature, humidity levels, microbiological control, and food quality control.

### 4.2 Bioactive agents used in food packaging

Scientific and technological progress is integral to successful production, and the transition to a higher level of consumers. The use of microbiological synthesis and the ability to control organic components of bio-additives has become a promising development in the food industry. Thus, antimicrobial peptides (AMP) are a viable choice for food preservation. So, Bi et al. [89], obtained peptides from the hydrolysate of the halibut inside. Sm-A1 (GITDLRGMLKRLKKMK), a peptide of 16 amino acids, has demonstrated outstanding antibacterial activity against both gram-positive and gram-negative bacteria, damaging the integrity of the cell membrane. It is important to note that Sm-A1 has been successfully loaded into hydroxyl-rich polyvinyl alcohol (PVA) and chitosan to improve antibacterial activity and the effect of biofilm inhibition. It has been established that PVA/chitosan+7.5 % Sm-A1 hydrogel can act as an effective additive for food packaging.

Having analysed statistical data on the attitude to dietary supplements in food film, films with increased antioxidant and antibacterial properties are used in the region of East Asian countries. Such an element as Zn has an antimicrobial effect and can also be used as a disinfectant component. Therefore, in the work of Jayakumar et al. [90], composite films based on starch and polyvinyl alcohol containing zinc oxide nanoparticles and phytochemicals prepared by casting from a solvent have increased water barrier, mechanical and antimicrobial properties, as well as unique physicochemical properties and the ability to indicate pH.

Biological synthesis attracts more entrepreneurs and production owners due to the lack of inorganic components for developing biologically active materials. Using such materials in food film, as established by the Environmental Protection Agency, can increase the resistive properties against various bacterial cultures. Thus, the study of Yu et al. [91] was aimed at developing new multifunctional packaging materials based on soy protein with the inclusion of cellulose nanofibrils (CN), *Cedrus deodara* pine needle extract (PNE) and lactic acid. The results showed that the inclusion of CN in the composite increased its tensile strength due to the effect of filling CN. The addition of CN and PNE significantly improved the opacity of the films. Moreover, the films containing PNE showed strong antioxidant activity and significantly improved the antimicrobial effect on foodborne pathogens of various bacterial cultures.

Due to the environmental situation in the modern world, the development of biodegradable packaging is an important task. Consumers from all over the world, as well as the Greenpeace organisation supporting them, actively advocate for the development of this direction, in connection with which many studies have been conducted on the development of such environmental packaging. Thus, the study carried out by Sganzerla et al. [92] aimed to develop an innovative biodegradable packaging with antimicrobial and antioxidant properties, functionalised by the by-product of *Acca sellowiana* (*A*. *sellowiana*) waste. The addition of preparation positively affected physicochemical, morphological, antioxidant, and antimicrobial properties in the postharvest conservation of apples. The high concentration of bioactive compounds in the films with antimicrobial capacity contribute to protect food products from microbial spoilage. Namely against *E*. *coli, Salmonella typhimurium* (*S*. *typhimurium*) and *Pseudomonas aeruginosa* (*P*. *aeruginosa*), which can help extend the shelf life of the target foods. As a result, it can be concluded that, in each territory of the world market of the food industry, it is necessary to deal with various external and internal factors. Casalini et al. [93] analysed the antioxidant and antimicrobial activity of essential oils of thyme, cinnamon, and oregano essential oils, incorporated into nanocellulose matrix as an active packaging solution for the shelf-life extension of food products. To thoroughly evaluate the system's effectiveness, the *in vitro* experiments were performed to test their activity against *S*. *aureus* and *E*. *coli* bacterial strains and directly on packed fresh raspberries. The obtained results revealed that this approach constitute a promising technology to improve shelf-life of raspberries.

## 5 Antimicrobial agents in food packaging applications

Antimicrobial agents can be applied to foods systems through two ways i) by direct addition to the food products, and ii) by an indirect system incorporating the antimicrobial compounds into the packaging materials [94]. The direct addition of the antimicrobial compounds into the foods and food products can reduce the effectiveness of the antimicrobial activity of the compounds due to the interaction between the compounds and food components [95,96]. Indirect contact can solve this issue because the compounds diffuse from the packaging material into the food surface to protect the food from unwanted spoilage and pathogenic bacteria. The efficiency of the indirect system depends on the release kinetics of compounds from the packaging material and relies on the properties of compounds incorporated into the packaging material [95,97], and the nature of the base matrix used in the packaging formulation. Table 1 shows some examples of phytochemicals and bioactive constituents efficiently used as antimicrobial agents in food industry.

**Table 1:** Overview of application phytochemicals and bioactive constituents as antimicrobial agents used in food packaging.

Phytochemicals and bioactive constituents	Packaging approach	Antimicrobial effects	Food products	References
Tea polyphenols (TP)	Incorporation of tea polyphenol to polyvinyl alcohol (PVA)	Gradual increase in bacteriostatic rates of the bacteria (*E*. *coli, S. aureus*)*,* and molds *(B. cinerea, Rhizopus*)	Strawberries	[98]
Cinnamaldehyde (CI) or TP	Incorporation in polylactic acid (PLA), polybutylene adipate (PBAT) and starch blends by extrusion technique	Effective inhibition of the growth of *E*. *coli* and *S*. *aureus* at 4 °C, reducing 3.6 and 4.1 log CFU/g on day 10, respectively	Meat analogues	[99]
TP	Incorporation in poly (vinyl alcohol)/ethyl cellulose nanofibrous films with the blending electrospinning technique	Inhibition rates of 15.84–88.39 % against *E*. *coli* and 21.10-.69 % against *S*. *aureus*	Meat	[100]
Grapefruit seed extract (GSE) and zinc oxide nanoparticles (ZnO)	Addition of the compounds to a blend film of poly(lactide) (PLA) and poly (butylene adipate-*co*-terephthalate) (PBAT)	Reduction of microbial growth around 2.02, 2.82, and 2.06 log CFU/g for *E*. *coli*, and 1.59, 1.77, and 1.47 log CFU/g for *S*. *aureus* in onion, cabbage, and carrot after 7 days of storage at 10 °C, respectively	Fresh-cut vegetables (onion, cabbage, and carrot)	[101]
Rambutan peel extract and cinnamon oil	Rambutan peel extract and cinnamon oil incorporated into cassava starch and whey protein blend films	Reduction of microbial growth with TVC up to 5.1 log cfu∙g^−1^ in salami at 10 days.	Slices of salami	[102]
TP	Vacuum package combined with tea polyphenols (V + TP)	TVC of V + TP group reach 5.82 log CFU/g, significantly lower control	Weever (*Micropterus salmoides*)	[103]
Polyphenol-rich kiwi peel extracts and silver nanoparticles	Sodium alginate-based nanocomposite films enhanced by polyphenol-rich kiwi peel extracts bio-reduced silver nanoparticles	Excellent antibacterial activities against *S*. *aureus* and *E*. *coli.*	Fresh cherries	[104]
Eugenol (EUG), carvacrol (CAR) and *trans*-anethole (ANT)	Cellulose (CE) and polypropylene (PP) pillow packages inserted with eugenol, carvacrol and *trans*-anethole	Reductions of microbial growth of −1.38, −0.91 and −0.93 (Δlog CFUg^−1^), respectively, with CAR, EUG and ANT_CE packages	Organic ready-to-eat iceberg lettuce	[105]
TP	Chitosan/halloysite nanotubes/tea polyphenol composites	Maximum rate of blueberry decay in the CS/HNTs-TP group was only 59 %, lower than control group, 87 %.	Fresh blueberries	[106]
Olive mill wastewater phenols capping ZnO nanoparticles	Carboxymethylcellulose incorporating olive mill wastewater phenols capping ZnO nanoparticles	Decrease in the weight loss and the rotting ratio of fresh strawberry, and at least 4 days of shelf life extension under 25 °C	Fresh strawberry	[107]
Pomegranate (*Punica granatum* L.) peel extract	Nano-encapsulation with alginate nanospheres to PPE	Total count value of 6.5 log CFU/g after 14 days storage, within the acceptable range of ICMSF	Chicken meat	[108]

Legend: *E. coli: Escherichia coli; S. aureus: Staphylococcus aureus; B. cinerea: Botrytis cinerea;* CFU: colony-forming unit; V + TP: vacuum package combined with tea polyphenols; TVC: total viable counts; EUG: eugenol; CAR: carvacrol; ANT-CE: *trans*-anethole inserted into cellulose; ICMSF: International Commission on Microbiological Specification for Foods.

Consumers increasingly prefer natural over synthetic products in recent years. As a result, naturally derived antimicrobial compounds are becoming essential in antimicrobial packaging since they are regarded to provide a lesser danger to consumers. These natural substances are considered safer than the synthetics and are believed to reduce some safety issues. Tea polyphenol potentially reduces the risk of high blood pressure and cholesterol concentration [109], cancer [110], diabetes, and cardiovascular disease [111]. Major polyphenolic compounds in green tea include epigallocatechin-3-gallate (EGCG), epigallocatechin, epicatechin-3-gallate and epicatechin, gallocatechin and gallocatechin gallate [111]. Carvacrol has been reported to have anti-cancer properties in preclinical breast, liver, and lung carcinoma models, acting on proapoptotic processes [112]. Another way is zinc oxide nanoparticles, which can help the human immune system and metabolism function [113]. When applying the compounds in the packaging with an indirect system, each phytochemical has distinct levels of antimicrobial activity to inhibit spoilage bacteria in food products. Concentration plays a vital role in microbial inhibition, in which the higher concentration of the chemical compounds increases the inhibition of microbial growth. However, the release rate of the compounds from the materials must be considered because a fast release rate produces a rapid increase in compound release and concentration in the packaging system. The high concentrations might influence the consumers' perceptions and degrade earlier than the bacterial growth. Slowing down the release of the compounds benefits in balancing the bacterial growth and concentration of compounds in the packaging system so that food spoilage can be inhibited for longer.

### 5.1 Encapsulation technique

Encapsulation is an advanced method to incorporate natural compounds in food packaging material. The natural compounds-entrapped material function as the major ingredients of functional food formulations, and effectively integrates into food packaging systems, protecting the biological activity and structural integrity of other components by preventing lipid oxidation, inhibiting bacterial growth, and thus extending food product shelf life. Kim et al. [101] investigated the encapsulation of grapefruit extract and zinc oxide nanoparticle (ZnO) to polylactic acid (PLA) and polybutylene adipate-*co*-terephthalate (PBAT) film. The developed film was applied to onion, cabbage, and carrot and showed a strong antimicrobial activity by reducing the 2.02–2.82 log CFU/g for *E. coli* and 1.47–1.77 log CFU/g for *S. aureus*, lower than control samples after seven days. Besides fresh fruit, nanoencapsulation is effectively applied in meat products. Rahnemoon et al. [108] encapsulated the pomegranate (*Punica granatum*) peel extract with alginate nanospheres applied to chicken meat. The result shows that the nano-encapsulated PPE coating can extend the shelf life of the chicken to 14 days of storage, where the microbial population reach 6.5 log CFU/g, which is still within the acceptable range to be consumed. Furthermore, incorporating two compounds in a film polymer can better inhibit bacterial growth. Wang et al. [99] incorporated cinnamaldehyde and tea polyphenols with PLA, PBAT, and starch film, which inhibited the growth of *E. coli* and *S. aureus* with a reduction by 3.6 and 4.1 log CFU/g on day 10.

### 5.2 Parameters affecting the release of bioactive compounds

The encapsulation of antimicrobial compounds in film/coating polymer leads to a controlled release of compounds from the polymer to the food product environment. A variety of release mechanisms are reported in the scientific literature. Mousavi et al. [114] incorporated epsilon-poly-l-lysine in fish gelatin-chitosan composite films. They showed that the moisture or water molecules from food products diffuse into the film, generating the polymer matrix swollen and then releasing the compounds from the coating composite. The slower release of compounds could be caused by the concentration-dependent diffusion of the compounds from nanoparticles into the food surface [115]. This mechanism is called swelling-induced release. Another mechanism is reported by Yang et al. [116], incorporating sugarcane bagasse to nanocellulose/nisin hybrid films. The release occurs due to the hydrogen bond between amino groups of nisin and hydroxyl groups of nanocellulose slowing the release of nisin. Maliha et al. [117] reported that the compounds can be entrapped in the fibres or film polymer. The insolubility of the polymer could limit the release of the compounds out of the packaging polymer. It also needs to be considered that external factors, e.g., temperature, humidity, light, and internal factors, e.g., pH, humidity, could influence the effectiveness of released compounds from packaging polymer.

A novel way to control the release of chemical compounds is reported by Bahmid et al. [66], who investigated the release of chemical compounds from natural resources. The release of allyl isothiocyanates is controlled by ground mustard seeds’ properties, e.g., fat content and particle sizes. Before the release of the compounds, the ground seeds absorb the moisture or water molecule to start the formation of AITC and then its release into the packaging system. Bahmid et al. [118] also developed an antimicrobial film by incorporating the ground mustard seeds into the cellulose acetate to sustain the release of the compounds. The release of compounds starts with the absorption of water molecules diffusing to the film, in which the water molecules trigger the AITC formation, diffusion and release from the packaging matrix. The film was evaluated on ground beef products, where shelf life was increased by 4.5 days. Additionally, the application of bioactive constituents can also be combined with another packaging concept, such as vacuum packaging.

## 6 Role of phytochemicals and active compounds used in food packaging. Effect on food shelf life

Food packaging containing phytochemical and bioactive compounds regarded as antioxidants, are of utmost importance to scavenge oxygen radicals. However, some active compounds used in food industry as preservatives or added to the packaging polymer, may probably have side effects on human health, e.g., the carcinogenic effects of butylated hydroxyanisole (BHA) and butylated hydroxytoluene (BHT) and the allergies caused by benzoic acid and sulphites, are reported in the literature. Increasing interest to find and replace the synthetic antioxidants by natural antioxidants obtained from bioresources including leaves, spices, herbs, and shrubs, are categorised as safe for human consumption. Table 2 shows a variety of phytochemicals and bioactive constituents obtained from different plants reported recently.

**Table 2:** Overview of antioxidant role of phytochemicals and active constituents used in food packaging.

Phytochemicals and bioactive constituents	Antioxidant role	Food packaging type and main findings	Reference
Chitosan and mangosteen (*Garcinia mangostana* L.) rind powder	Inhibition of the increase in the peroxide value and thiobarbituric acid reactive substances of soybean oil	Coating ∼ the antioxidant activity released from CS-MRP films inhibited the decomposition of hydroperoxides into secondary oxidation products.	[122]
Tea polyphenol (TP)	Reduction of the DPPH radical and the extent of the reaction mainly depends on the hydrogen donating ability of the antioxidants.	Coating ∼ scavenging capacities significantly increased in the presence of TP.	[119]
Tannin	Quick reaction with the DPPH radical (fast kinetic behaviour) taking around 40 min to reach the steady state.	Coating ∼ The functional activity of the active agent incorporated into a film matrix.	[123]
Chitosan and tea polyphenols catalysed by laccase	31 % of DPPH radical scavenging rate of chitosan film.	Coating ∼ laccase crosslinking enables the formation of covalent bonds	[124]
Polyphenols from myrtle berries extract (MBE)	The redox properties pf phenolic compounds scavenge free radicals and act as reducing agents.	Film under the lid of the bottle ∼ Increasing MBE concentration in the alginate matrix increased the release rate in the food simulant	[120]
Chitosan/potato peel polyphenols nanoparticles	Increasing to respectively 59.6 % and 54.8 % of DPPH radical and ABTS radical cation scavenging activities	Coating an extended release of PP for 1440 min described with Fick's diffusion model accurately predicted the release kinetics of PP	[121]
Chinese chive (*Allium tuberosum*) root extract (CRE)	Increasing up to 57.38 % of DPPH radical and ABTS radical cation scavenging ability.	Film samples were sealed with line rope on the top of glass bottles ∼ Increasing total phenolic contents increases concentration of CRE in CS-CRE films.	[125]
Gliadin/phlorotannin nanoparticles (GPNP)	Increasing the GPNP concentration and extending the release time increase DPPH radical and ABTS radical cation scavenging capacity	Coating ∼ release of phlorotannin mainly occurred in the first 6 h with 60 % of the total release and after 14 h, the release rate reached stable.	[126]
Spent coffee ground	High scavenging radicals activity for DPPH radical than ABTS radical cation.	Coating ∼ gelatine contributes to the antioxidant activity of the films.	[127]

Legend: CS-MRP: chitosan incorporated with mangosteen rind powder films; TP: tea polyphenols; DPPH: 1,1-diphenyl-2-picrylhydrazyl radical; MBE: myrtle berries extract; PP: potato peel polyphenols; ABTS: 2,2′-Azinobis-(3-Ethylbenzthiazolin-6-Sulfonic Acid; CRE: chive root extract; CS-CRE: chitosan incorporated with chive root extract films.

**Fig. 4:**
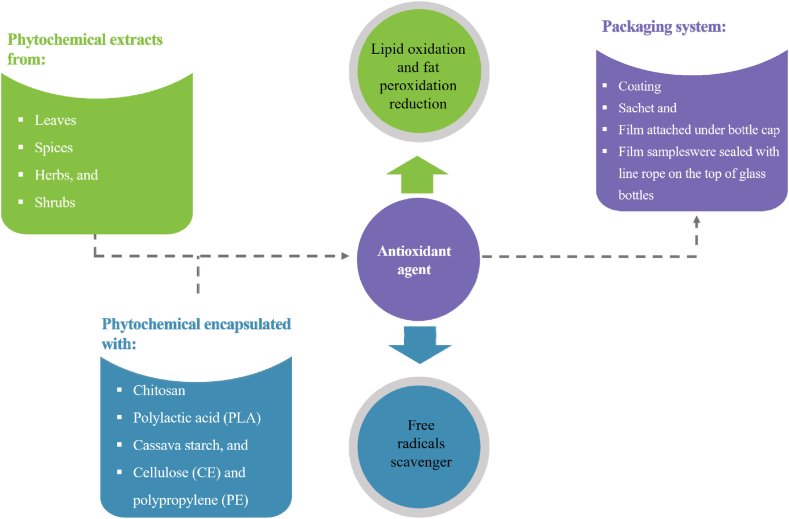
Process stage of phytochemicals and active compounds extracted, encapsulated, functioned, and implemented in food packaging.

Currently, the scientific community is very interested in the development of smart packaging systems (Fig. 4), being the smart (active and intelligent) compounds encapsulated and incorporated into the coating systems, with a release mechanism whose kinetic depends on several parameters (storage time and temperature, food nature, among others).

Phytochemicals and active compounds can also be applied as an antimicrobial packaging system. One of the most critical tasks of food packaging is to slow down the natural processes that cause food to spoil. Antioxidants and antimicrobials are often included in food packaging materials during manufacturing and released into the packed food through a controlled diffusion method. Antioxidants are used in food packaging to attenuate lipid oxidation and protein denaturation [128,129]. Unlike conventional packaging, which must be completely inert, active packaging is intended to interact with the ingredients and/or the environmental conditions to fulfil functions other than being a barrier to outside influences [130]. Food contact (active upon contact) materials and articles have been created with “active” components that release targeted substances into the packed food or absorb chemicals from the packaged food and/or the environment surrounding the food. Wieczyńska and Cavoski [105] developed a packaging film of cellulose and polypropylene inserted with eugenol, carvacrol, and *trans*-anethole, where this film was attached to the upper part of the packaging to avoid direct contact between the packaging material and foods products. This system shows an increase in the release of compounds and antioxidant activity in the packaging headspace. Phlorotannin from nanocomposite films is also released under the influence of environmental conditions, occurring in the first 6 h with 60 % of the total release [126].

Phytochemicals as antioxidants inhibit the reaction of oxidation led by free radicals, e.g., superoxide, singlet oxide, hydroxyl radicals, and peroxyl radicals; thereby, cell damage can be delayed and prevented. Phytochemicals, e.g., tea polyphenol, diminish DPPH radicals, and the intensity of the reaction is mainly determined by the antioxidants' capacity to donate hydrogen [119]. Scavenging activity rate has been reported differently, depending on the type of compounds. Chollakup et al. [102] found a quick release of DPPH radical scavenging activity after 35 min, in which the release depends on the starch type and concentration. d-α-tocopheryl polyethylene glycol, succinate, and silicon dioxide nanoparticles have 31.58 % DPPH radical scavenging activity at 5 mg/mL [89]. Chen et al. [124] also observed 31 % of DPPH radical scavenging activity with chitosan and polyphenols. The most interesting is that chitosan/potato peel and polyphenols nanoparticles have a higher performance to DPPH and ABTS radical scavenging activities, reaching 54.8–59.6 % [121]. Free radicals can oxidise lipids and other food ingredients when present in food packaging, which can cause food to deteriorate and lose quality. Mechanisms of scavenging reactive oxygen and nitrogen free radical species reduce localised oxygen concentrations, reduce molecular oxygen's oxidation potential, metabolise lipid peroxides to non-radical products, and chelate metal ions to prevent the generation of free radicals. The scavenging activity was also reported to be influenced by the free amino group scavenging free radicals to yield stable macromolecule radicals [89,124]. Thermally produced free radicals in packaged meals and antioxidant chemicals inside the packaged food would contribute electrons to the free radicals, completing the outer shell ions of the radical species.

Lipid peroxidation is one of the most critical processes contributing to food degradation and microbial growth. Lipid peroxidation is a cause of food degradation, resulting in loss of functional and nutritional value. Quiles-Carrillo et al. [131] showed that adding gallic acid (GA) with varying concentrations to the Bio-HDPE matrix considerably increased the films' UV stability. The UV stability could be maintained with the lowest GA level for 10 h, protecting materials from UV light and preventing lipid oxidation in foods. Furthermore, phytochemical compounds also contribute to preventing lipid peroxidation, a major cause of food deterioration leading to a loss of functional properties and nutritional value. Polyunsaturated fatty acids that have been oxidised may promote ageing and cancer. A self-catalytic free radical chain reaction occurs in the main route of lipid peroxidation. Nadeem et al. [132] found that although margarine had a greater content of polyunsaturated fatty acids, phenolic compounds from chia oil effectively reduced lipid peroxidation in supplemented margarine. Chia oil's phenolic substances effectively inhibited lipid peroxidation, as evidenced by the lower peroxide value, while flavour score and peroxide value were strongly correlated.

The release of phytochemicals and bioactive compounds onto food surface needs to be controlled by incorporation or encapsulation to improve antioxidant activity [133]. Cheikh et al. [120] investigated the antioxidant activity of polyphenols from myrtle berries encapsulated with alginate bio nanocomposite films, while Ma et al. [121] reported the antimicrobial activity of encapsulated polyphenols with chitosan/potato peal nanoparticles. Incorporating the compounds into the coating material generates a sharp release due to a good solubility between the polyphenols and food simulants and because free myrtle berries extract molecules on the film's surface. The solubility of the polyphenols in hydroxypropyl methylcellulose (HPMC) in methyl cellulose-based films triggered the release of phenols into a high-humidity environment. Furthermore, increasing myrtle berry extract concentration and alginate matrix can also increase the antioxidant release in food simulant/food products. The starch alginate matrix containing more hydroxyl groups and partially dissolved in water resulted in a larger release of phenolic chemicals [102]. The water exposing the hydroxyl groups in the starch facilitates swelling to polymer matrices and increased migration. Furthermore, the interaction between protein and polyphenol decreased free polyphenol and resulted in less phenolic compound released into the water [102]. The food polymer packaging can behave as a polymer matrix or carrier, allowing the oxygen-scavenging active ingredient to leach into the oxygen-sensitive packaged food.

To improve the oxidative stability of soybean oil in the food business, chitosan and mangosteen (*Garcinia mangostana* L.) rind powder film packing successfully reduced the growth in peroxide value and thiobarbituric acid reactive chemicals during storage [122]. However, environmental variables such as light, oxygen, free radicals, and metals can promote lipid peroxidation. The most often employed antioxidants for this function are phenolic compounds and organic sulphides. As an example, consider hindered phenol [134]. When nearby carbon atoms' linked hydrogen is substituted with transition metals, a hindered phenol molecule is formed, which resists the oxidative breakdown of polymers like rubber. Natural antioxidants can be included in polymer plastic packaging to initially influence the packaging, either stabilise the packaging against UV degradation or being released into food to stabilise the food against oxidation. Whereas natural antioxidants would comply with packaging regulations, food packaging legislation would have been satisfied in the second case pursuant to community or national requirements regulation (EC) 450/2009 [135].

Researchers, manufacturers, and the food industry sector have been focused on finding safe and effective strategies to maintain the quality and prevent food spoilage. The use of non-thermal methods on food preservation opens a new market segment for consumers [136]. Manzoor et al. [137] found that the mango peel extracts inhibited protein oxidation by reducing carbonyl levels, improving chicken meat content, and enhancing consumer acceptability. The scientific value of cherries added to meat and their health advantages was also discussed by Brodowska et al. [138]. Rabadán et al. [139] investigated the feasibility of replacing lamb fat and starch with seeds, nut oils, and defatted flours. There was a positive consumer response to lamb meat burgers with improved health benefits.

Oxidative processes decrease food quality, shelf life and nutritional content. Antioxidants and phytochemicals are among the most effective ways to control food oxidation. Natural phenolic compounds have been shown to reduce oxidation, foodborne pathogens, and bacterial spoilage in meat [140]. Vital et al. [141] studied the effects of rosemary and oregano essential oils on beef steak quality parameters. These essential oils reduced lipid oxidation by 46.81 % and improved consumers' acceptability and perceptions of odour and flavour. Consumers and stakeholders responded positively to adding phytochemicals to replace nitrites in processed meat [142]. Some phytochemical compounds have chelating properties, inhibiting transition metals from acting as oxidising agents [143].

Research has recently focused on phytochemicals naturally occurring in plants because of their wide range of health benefits and consumer acceptance. Akcan et al. [144] evaluated the impact of hawberry phenolic based on pork meat. They reported the significant inhibition of lipid oxidation in addition to improving consumer satisfaction with meat's odour. In addition to their use as additives, polyphenols have a crucial role in preserving meat products [145]. The food quality and shelf life of different food sources as affected by phytochemicals are presented in Table 3.

**Table 3:** The effect of phytochemicals on food quality and shelf life.

Phytochemical	Food source	Results	Reference
Catechin, quercitrin, and epicatechin	Kiwifruit extract	Prevents lipid oxidation in beef without affecting its sensory characteristics.	[146]
Phenolic compounds	Rosemary	Lamb meat was effectively protected from lipid oxidation	[147]
Polyphenols	Apple peel	Inhibition of fish oil oxidation	[148]
Carvacrol, thymol, *p*-cymene and γ-terpinene	*Origanum compactum* essential oil	Preserving field and stored rice by growth inhibition of the *Bipolaris oryzae* and *Fusarium* spp.	[149]
Phenolic compounds	*Hibiscus sabdariffa* extracts	Providing extended shelf life in beef due to inhibition of foodborne pathogenic bacteria.	[150]
Green tea catechins	Green tea	Providing the inhibition of microbial deterioration and lipid oxidation in hamburger.	[151]
Green tea catechins	Green tea	Maintaining the shelf-life of pork sausages by inhibiting microbial growth.	[152]
Phenolic compounds	*Syzygium aromaticum* (L.), *Cinnamomum cassia* (L.), and *Origanum vulgare* (L.) extracts	Providing inhibitory effects on microbial growth and lipid peroxidation in raw chicken meat.	[153]
Phenolic compounds	Rosemary and cloves extract	Preventing microbial activity, decreasing lipid oxidation, conserving sensory properties, and increasing raw chicken meat's shelf life.	[154]
Phenolic compounds	Barley husks	Improving salmon fish oxidative stability and reducing lipid hydrolysis.	[155]
Flavonoids,	Fruit of hagimit	Aqueous hagimit extract was found to extend the shelf life of mature green tomatoes by 120 %.	[156]
Curcumin and Piperine	Black pepper and Turmeric	Piperine, curcumin, and honey nanoemulsions inhibited over 80 % of *Candida* strains.	[157]
α-Tocopherol	Winter wheat	Fish oil lipid peroxidation was significantly inhibited by wheat extracts.	[158]
Phenolic subclasses, tocopherols, and ascorbic acid	Common hawthorn (*Crataegus monogyna* Jacq.)	Oxidative damage to lamb proteins was significantly inhibited.	[159]
Phenolic compounds and betalains	*Amaranthus* leaf extract	Improved the shelf life of fish/chicken from 3 to 12 days due to antibacterial properties, protecting from UV light, and reducing water solubility.	[160]
]Polyphenol compound (catechins)	Matcha green tea powder	Biscuit quality and consumer acceptance were positively affected by these phytochemicals.	[161]

## 7 Limitations and overcoming mechanisms in phytochemicals and bioactives in packaging

Phytochemical and bioactive compounds have a high potential to be implemented in the packaging system. Encapsulation methods, natural sources, and combination with another packaging system, e.g., vacuum packaging, result in the effectiveness of the compounds as antioxidant and antimicrobial agents. The release of compounds into the food surface or packaging headspace generates an interaction between the compounds with microorganisms growing the foods and free radicals found in the foods. However, some considerations are still challenging in implementing phytochemical and bioactive packaging systems; First consideration is the degradation of compounds during storage. Bahmid et al. [162] reported that after an active compound is released from the film material to the packaging headspace or food simulants, the compounds would be degraded in the packaging headspace or the foods. Many factors, such as temperature, the presence of moisture and reaction with other food components, could influence the degradation. The degradation could be controlled by altering the polymer composition [163] and increasing the thickness of the films [118], which could need further investigation to overcome the issue of degradation of the compounds inside the packaging system, so the effectiveness of the compounds could be optimised. The second consideration is consumer acceptability. The consumer is the final target, so the food shelf life needs to be extended. Using bioactive compounds inside the packaging system could generate an aroma from the compounds volatilised once released from the packaging materials [164]. The volatile compounds might affect the willingness of consumers to buy products. Other sensory effects could be potentially present, such as colouring change. It is important to investigate further the concentration limit for each phytochemical compound to be acceptable in the packaging system. Lopes et al. [165] evaluated the sensory allyl isothiocyanate content on Brazil peanuts, where 0.5–2.5 μL/L is the acceptable concentration of compounds in the product packaging. Furthermore, the complexity of the mechanisms in food packaging is also a challenge. Different foods and chemicals might have other mechanisms and effectiveness of the packaging system in the extension of the shelf life of a food product, causing a further investigation before the packaging is implemented in a product.

## 8 Concluding remarks

The manuscript discusses intensely and in a comprehensive way the importance of phytochemicals and bioactive compounds in food field. Consumers' demand for safe ingredients and concerns over the harmful effects of synthetic additives have prompted food manufacturers to seek safer alternative solutions. Also, consumers increasingly prefer natural over synthetic products in recent years. As a result, naturally derived antioxidant and antimicrobial compounds are becoming essential in active packaging materials since they are regarded to provide a lesser danger to consumers and simultaneously increase the food shelf-life. Smart food packaging containing phytochemicals, found in many plants, vegetables, fruits, and herbs, emerged as the alternative solutions against the nocive nature of synthetic additives. Plant extracts can be added to packaging products and incorporated into films given them high antibacterial and antioxidant capacity. The volatile compounds might affect the willingness of consumers to buy products, and other sensory effects could be potentially present, such as colouring change. Therefore, in each territory of the world market of the food industry, it is necessary to deal with a variety of external and internal factors; therefore, for consumers of all levels, phytochemicals and bioactive compounds can be applied as an additional element included in food packaging.

## Funding

This research was funded by FCT-10.13039/501100001871Fundação para a Ciência e a Tecnologia through the CQM Base Fund - UIDB/00674/2020, and Programmatic Fund - UIDP/00674/2020, by 10.13039/100013276Interreg MAC 2014–2020 Cooperación territorial through AD4MAC project (MAC2/1.1b/350), and by ARDITI-Agência Regional para o Desenvolvimento da Investigação Tecnologia e Inovação, through the project M1420-01-0145-FEDER-000005 - Centro de Química da Madeira - CQM+ (Madeira 14–20 Program). The authors also acknowledge FCT and Madeira 14–2020 program to the Portuguese Mass Spectrometry Network (RNEM) through PROEQUIPRAM program, M14-20 M1420-01-0145-FEDER-000008).

## Ethics statement

Review and/or approval by an ethics committee was not needed for this study because this is a systematic reviews paper.

## Data availability statement

No data was used for the research described in the article.

## CRediT authorship contribution statement

**Shahida Anusha Siddiqui:** Visualization, Resources, Methodology, Investigation, Formal analysis, Data curation, Project administration, Supervision, Validation, Writing – original draft, Writing – review & editing. **Sipper Khan:** Validation, Conceptualization. **Mohammad Mehdizadeh:** Writing – original draft. **Nur Alim Bahmid:** Writing – review & editing, Writing – original draft. **Danung Nur Adli:** Writing – review & editing, Writing – original draft, Methodology. **Tony R. Walker:** Validation, Supervision. **Rosa Perestrelo:** Validation, Investigation. **José S. Câmara:** Methodology, Writing – review & editing.

## Declaration of competing interest

All the authors declare that they have no known competing financial interests or personal relationships that could have appeared to influence the work reported in this paper.
