# Corrigendum to “Phytochemicals and bioactive constituents in food packaging - A systematic review” [Heliyon 9(11) (November 2023) e21196]

**DOI:** 10.1016/j.heliyon.2023.e22184

**Published:** 2023-11-14

**Authors:** Shahida Anusha Siddiqui, Sipper Khan, Mohammad Mehdizadeh, Nur Alim Bahmid, Danung Nur Adli, Tony R. Walker, Rosa Perestrelo, José S. Câmara

**Affiliations:** a Technical University of Munich Campus Straubing for Biotechnology and Sustainability, Essigberg 3, 94315, Straubing, Germany; b German Institute of Food Technologies (DIL e.V.), Prof.-von-Klitzing Str. 7, 49610, D-Quakenbrück, Germany; c Tropics and Subtropics Group, Institute of Agricultural Engineering, University of Hohenheim, 70593, Stuttgart, Germany; d Department of Agronomy and Plant Breeding, Faculty of Agriculture and Natural Resources, University of Mohaghegh Ardabili, Ardabil, Iran; e Ilam Science and Technology Park, Iran; f Research Center for Food Technology and Processing, National Research and Innovation Agency (BRIN), Gading, Playen, Gunungkidul, 55861, Yogyakarta, Indonesia; g Agricultural Product Technology Department, Universitas Sulawesi Barat, Majene, 90311, Indonesia; h Faculty of Animal Science, University of Brawijaya, Malang, East Java, 65145, Indonesia; i School for Resource and Environmental Studies, Dalhousie University, Halifax, Nova Scotia, B3H, 4R2, Canada; j CQM – Centro de Química da Madeira, Universidade da Madeira, Campus da Penteada, 9020-105, Funchal, Portugal; k Departamento de Química, Faculdade de Cîencias Exatas e da Engenharia, Universidade da Madeira, Campus da Penteada, 9020-105, Funchal, Portugal

In the original published version of this article, there is a mistake in Fig. 1 since one of the fields is empty (the right green box), without the corresponding information. The authors provided correct version for Fig. 1 [The correct version of Fig. 1 can be found below and in the Amended figure folder] and they apologize for the errors. Both the HTML and PDF versions of the article have been updated to correct the errors.

**Fig. 1.**

**Figure:**
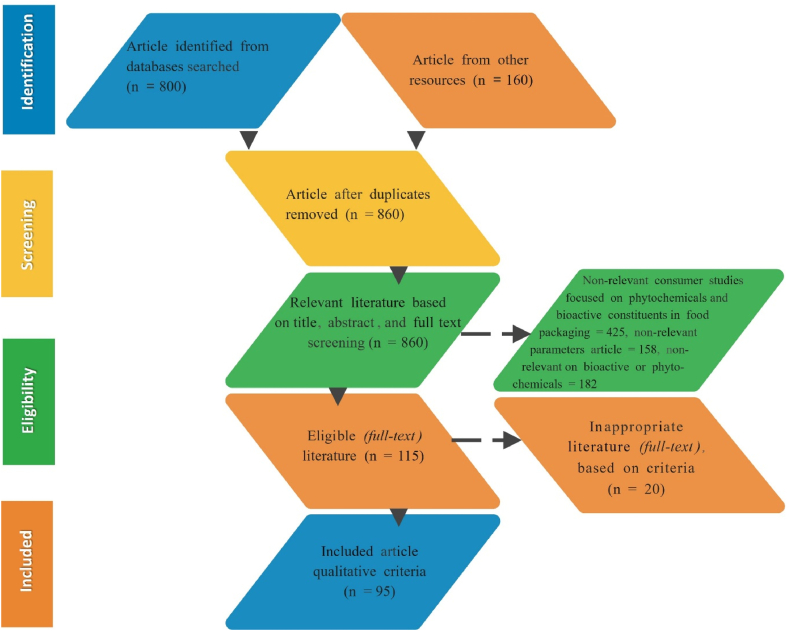

## Declaration of competing interest

All the authors declare that they have no known competing financial interests or personal relationships that could have appeared to influence the work reported in this paper.

